# Identification of a Novel Subtype‐Derived Prognostic Signature Based on Nucleotide Metabolism‐Related Molecular Classification for Predicting Lung Adenocarcinoma Prognosis

**DOI:** 10.1155/ijog/4944325

**Published:** 2026-10-02

**Authors:** Yue Shi, Shanshan Li, Lin Zhao, Yali Li, Fangyu Cai, Kun Wang, Naibo Xu, Benkun Liu, Fucheng Zhou, Yue Cui, Changguo Shi, Xue Bai, Jian Zhang

**Affiliations:** ^1^ Department of Thoracic Surgery Harbin Medical University Cancer Hospital Harbin Heilongjiang China hrbmu.edu.cn; ^2^ Department of General Clinic Heilongjiang Provincial Hospital Harbin Heilongjiang China hewiki.com; ^3^ Department of Internal Medicine Harbin Medical University Cancer Hospital Harbin Heilongjiang China hrbmu.edu.cn; ^4^ Department of Radiation Physics Harbin Medical University Cancer Hospital Harbin Heilongjiang China hrbmu.edu.cn; ^5^ School of Basic Medical Sciences Harbin Medical University Harbin Heilongjiang China hrbmu.edu.cn; ^6^ Department of Oncology The Second Affiliated Hospital of Anhui Medical University Hefei Anhui China ahmu.edu.cn; ^7^ Department of Thoracic Surgery Jiamusi Cancer Hospital Jiamusi Heilongjiang China

**Keywords:** drug sensitivity, immune infiltration, immunotherapy, lung adenocarcinoma (LUAD), nucleotide metabolism, prognostic signature

## Abstract

**Background:**

Lung adenocarcinoma (LUAD) is a major subtype of nonsmall cell lung cancer with substantial clinical heterogeneity. Nucleotide metabolism‐related genes may contribute to tumor progression and immune microenvironment remodeling, but their prognostic value in LUAD remains incompletely defined.

**Methods:**

Bulk transcriptomic and clinical data from TCGA‐LUAD and GEO datasets were analyzed to define nucleotide metabolism‐related molecular subtypes and construct a subtype‐derived prognostic signature from subtype‐associated differentially expressed genes. The TCGA‐LUAD cohort was randomly divided into a training cohort and an internal validation cohort at a ratio of 4:1. Univariate Cox regression and LASSO regression analyses were performed exclusively in the training cohort to construct the prognostic signature, which was subsequently evaluated in the internal validation cohort and the independent external bulk‐transcriptomic GSE30219 cohort. Consensus clustering, survival analysis, ROC analysis, immune infiltration analysis, mutation analysis, predicted drug sensitivity analysis, and exploratory single‐cell transcriptomic characterization were also performed. In vitro assays were used to evaluate the functional relevance of IRX5 in A549 LUAD cells.

**Results:**

A total of 152 subtype‐associated differentially expressed genes were identified from pairwise comparisons among the three nucleotide metabolism‐related molecular subtypes, and a nine‐gene subtype‐derived prognostic signature consisting of SEMA3C, PTTG1, BARX1, CDCA5, TGFBI, MKI67, TSPAN7, GADD45G, and IRX5 was constructed. The signature stratified LUAD patients into high‐ and low‐risk groups with distinct overall survival and remained an independent prognostic factor. The risk score was associated with TP53 mutation frequency, immune cell infiltration patterns, cytokine‐ and exhaustion‐related scores, and predicted IC50 values for several anticancer agents. Single‐cell analysis revealed cell‐type–specific expression patterns of signature genes and potential intercellular communication features in the LUAD microenvironment. Functionally, IRX5 knockdown inhibited proliferation, migration, and invasion, promoted apoptosis, and induced G0/G1 cell‐cycle arrest in A549 cells.

**Conclusion:**

This study identified a subtype‐derived prognostic signature based on nucleotide metabolism‐related molecular classification with potential value for LUAD risk stratification and provided exploratory evidence linking signature genes to tumor immune features and cellular phenotypes.

## 1. Introduction

Lung cancer is one of the most common malignant tumors in the world, and its mortality rate ranks first among cancers; it accounts for approximately 21% of all cancer‐related deaths [1, 2]. Nonsmall cell lung cancer (NSCLC) is the most common subtype of lung cancer and accounts for approximately 85% of all lung cancers. NSCLC can be histologically divided into lung adenocarcinoma (LUAD), lung squamous cell carcinoma (LUSC), and large cell carcinoma, among which LUAD is the most common histological subtype [3–5]. Currently, the principal treatment modalities for lung cancer include surgery, chemotherapy, radiotherapy, targeted therapy, and immunotherapy, but each method has its limitations [6–8]. Owing to the high malignant potential of lung cancer, the 5‐year survival rates are approximately 14%–49% for Stages I–IIIA and < 5% for Stages IIIB–IV [3]. Cancer immunotherapy has emerged as a promising cancer treatment, but only a fraction of patients responds to treatment [9–11]. Therefore, the development and verification of novel markers for predicting the clinical prognosis of LUAD at an early stage are urgently needed to improve the survival rate of patients.

Nucleotides are fundamental building blocks of living organisms and essential substrates for nucleic acid synthesis and cell proliferation [12, 13]. Nucleotide metabolism is in a state of dynamic equilibrium, which is important for maintaining the normal physiological functions of cells [14, 15]. Recently, researchers reported that nucleotide metabolism constitutes the final and most crucial link in the chain of events that contribute to the spread of cancer [14, 16–18]. To achieve uncontrolled cell proliferation, tumor cells use the nucleotide metabolism pathway to synthesize DNA and RNA [19]. Recent research has demonstrated that aberrant nucleotide metabolism can dampen the normal immune response in the tumor microenvironment (TME). Targeting nucleotide metabolism also represents a new direction for the development of novel antitumor‐specific drugs [20–22]. Therefore, focusing on the reprogramming of nucleotide metabolism will provide new ideas for predicting prognostic outcomes in LUAD patients. Moreover, the clinical relevance of nucleotide metabolism‐related genes in predicting outcomes and guiding the application of chemotherapeutic strategies for LUAD patients remains unknown. Thus, exploring the characteristics and interactions of nucleotide metabolism‐related genes in LUAD is important.

In the present study, we downloaded transcriptome profiles, single‐cell transcriptome data, and relevant clinical information for LUAD patients from the TCGA and GEO databases. We used nucleotide metabolism‐related genes to define molecular subtypes and subsequently screened subtype‐associated differentially expressed genes for prognostic modeling in LUAD. For prognostic model development, the TCGA‐LUAD cohort was randomly divided into a training cohort and an internal validation cohort at a ratio of 4:1. Univariate Cox regression and least absolute shrinkage and selection operator (LASSO) regression were performed exclusively in the training cohort to construct a nine‐gene subtype‐derived prognostic signature consisting of SEMA3C, PTTG1, BARX1, CDCA5, TGFBI, MKI67, TSPAN7, GADD45G, and IRX5. The resulting signature was subsequently evaluated in the internal validation cohort and further validated in the external GSE30219 cohort. The GSE149655 single‐cell dataset was used separately for exploratory characterization of cell‐type–specific expression and intercellular communication and was not used for patient‐level prognostic validation. Subsequently, the signature was further explored for its associations with clinical characteristics, the TME, and computationally predicted drug‐response patterns. High‐risk group patients experienced poorer survival times compared with low‐risk group patients. This nine‐gene subtype‐derived prognostic signature may serve as a candidate biomarker for risk stratification in LUAD patients.

## 2. Materials and Methods

### 2.1. Data Collection and Processing

The transcriptomic data and relevant clinical data related to LUAD patients were downloaded from the TCGA database (https://portal.gdc.cancer.gov/projects/TCGA-LUAD) and the GEO database (GSE30219, https://www.ncbi.nlm.nih.gov/gds/?term=GSE30219). We also obtained single‐cell transcriptome data from the GEO database (Accession Number GSE149655) on four samples: two primary LUAD samples and two normal tissue samples. For TCGA‐LUAD, the expression data were based on FPKM values. For clarity, the TCGA validation subset was used for internal validation of the prognostic model; GSE30219 served as an independent external bulk‐transcriptomic validation cohort for evaluation of the fixed prognostic model, but not for validation of a prespecified absolute risk‐group cutoff, and GSE149655 was used only for exploratory single‐cell characterization rather than patient‐level prognostic validation. In addition, GSE135222 was used for exploratory evaluation of the nine‐gene subtype‐derived prognostic signature in an NSCLC cohort receiving immune checkpoint inhibitor therapy, whereas IMvigor210, a metastatic urothelial carcinoma cohort, was included only as an exploratory cross‐cancer evaluation and was not considered a direct validation cohort for NSCLC or LUAD.

To account for platform‐related differences while preserving the independence of the external validation cohort, TCGA‐LUAD and GSE30219 were preprocessed separately. For each cohort, the expression matrix was log2‐transformed and gene‐wise *Z*‐score standardized before model application. All feature selection, LASSO‐Cox regression and coefficient estimation were performed exclusively in the TCGA training cohort. The fixed coefficients derived from the TCGA training cohort were then directly applied to the independently preprocessed GSE30219 dataset to calculate patient‐level risk scores. The TCGA‐LUAD and GSE30219 expression matrices were not merged, and no cross‐platform batch‐correction procedure was applied.

### 2.2. Consensus Clustering and Identification of Subtype‐Associated Differentially Expressed Genes

Nucleotide metabolism‐related genes (relevance score > 20) were obtained from the GeneCards database (https://www.genecards.org). Consensus clustering was then performed in the TCGA‐LUAD cohort based on the expression profiles of these genes. The number and stability of the clusters were evaluated using the cumulative distribution function (CDF) and delta area curves, and *k* = 3 was selected as the optimal solution, yielding three molecular subtypes, C1, C2, and C3. Kaplan–Meier survival analysis and the log‐rank test were used to compare overall survival (OS) among the three molecular subtypes. Subsequently, pairwise differential‐expression analyses were performed using the “limma” package for C1 versus C2, C1 versus C3, and C2 versus C3, with *p* < 0.05 and fold change > 1.5 as the screening criteria. The intersection of the three pairwise differentially expressed gene sets yielded 152 subtype‐associated differentially expressed genes, which were used for subsequent functional enrichment and prognostic‐model construction.

### 2.3. Functional Enrichment Analysis

To investigate gene functions in each gene cluster, we used Metascape (http://metascape.org/gp/index.html#/main/step1) to perform Kyoto Encyclopedia of Genes and Genomes (KEGG) pathway and gene ontology (GO) functional enrichment analyses. Additionally, gene set enrichment analysis (GSEA) was performed using GSEA software (Version 4.3.2; Broad Institute) with the KEGG LEGACY gene set collection from the C2:CP collection of MSigDB Human v2022.1.Hs to assess pathway‐level differences between the high‐ and low‐risk groups of LUAD patients.

### 2.4. Establishment of a Subtype‐Derived Prognostic Signature

Before model construction, TCGA‐LUAD patients with complete survival information were randomly divided into a training cohort and an internal validation cohort at a ratio of 4:1 using a fixed random seed. All feature‐selection and model‐fitting procedures, including univariate Cox regression, LASSO regression, and estimation of the model coefficients, were performed exclusively in the training cohort.

The 152 subtype‐associated differentially expressed genes obtained from the intersection of the three pairwise subtype comparisons were first subjected to univariate Cox proportional hazards (PHs) regression in the training cohort, and genes with *p* < 0.05 were retained as prognostic candidates. Subsequently, LASSO‐Cox regression with an L1 penalty was used for further feature selection and to reduce model overfitting. The optimal penalty parameter (*λ*) was selected by cross‐validation at lambda.min, resulting in nine genes for construction of the subtype‐derived prognostic signature. The risk score for each patient was calculated as a linear combination of the standardized expression values of the nine genes and their corresponding LASSO coefficients, using the following formula: Risk  score = IRX5 × (−0.128648463) + GADD45G × (−0.077826735) + TSPAN7 × (−0.073307189) + MKI67 × 0.000163894 + TGFBI × 0.02372854 + CDCA5 × 0.035798743 + BARX1 × 0.089405586 + PTTG1 × 0.122970952 + SEMA3C × 0.135467087. For Kaplan–Meier stratification, the median risk score was calculated separately within each cohort (the TCGA training cohort, the TCGA internal validation cohort, and GSE30219) and used as a cohort‐specific cutoff; the training‐cohort median cutoff was not directly transferred to the validation cohorts. Kaplan–Meier survival curves and log‐rank tests were used to compare OS between the two groups. Time‐dependent receiver operating characteristic (ROC) curves and the area under the curve (AUC) were used to evaluate the prognostic performance of the signature. The cohort‐specific median was used only for dichotomization in Kaplan–Meier analyses, whereas time‐dependent ROC analyses were based on the continuous risk score and therefore did not depend on this cutoff.

The resulting nine‐gene subtype‐derived prognostic signature was subsequently evaluated in the internal validation cohort by directly applying the fixed coefficients derived from the training cohort, without additional feature selection or model refitting, and was further assessed in the external GSE30219 cohort. Thus, the validation cohorts were used to evaluate the fixed model coefficients and continuous risk score, whereas a prespecified absolute dichotomization threshold was not externally validated.

### 2.5. Immune Cell Infiltration Analysis

CIBERSORT is a method based on the input matrix of a gene expression file to accurately estimate the relative proportions of various immune cell subtypes in tissues. Here, we used CIBERSORT analysis to assess differences in the infiltration levels of various immune cells in distinct groups.

### 2.6. Construction and Evaluation of the Nomogram

After establishing the nine‐gene subtype‐derived prognostic signature, the corresponding risk score was integrated with clinical variables, including age, sex, overall stage, and the individual T, N, and M categories, to construct a nomogram for individualized survival prediction. Calibration curves were used to assess the agreement between the nomogram‐predicted and observed 3‐ and 5‐year OS probabilities. Although overall stage and the individual T, N, and M categories are clinically related, they were retained together because they provide staging information at different levels of granularity. Specifically, T, N, and M describe local tumor extent, regional lymph‐node involvement, and distant metastatic status, respectively, whereas overall stage provides an integrated summary of disease extent. Potential multicollinearity among all covariates included in the Cox model was further assessed using variance inflation factors (VIFs).

### 2.7. Single‐Cell Transcriptome and Cell–Cell Crosstalk Network Analysis

The quality control and preprocessing of the scRNA‐seq data were performed using the Seurat package. For initial cell filtering, cells with nFeature_RNA > 50 and percent.mt < 25 were retained for subsequent analyses. The raw expression matrix was normalized using the Seurat NormalizeData function. The top 2000 highly variable genes were identified using FindVariableFeatures, followed by principal component analysis (PCA) based on these variable genes. The first 30 principal components (PCs) were retained for downstream clustering and visualization. Unsupervised cell clustering was performed using the FindClusters function with a resolution parameter of 0.5, which was selected by considering both cluster stability and biological interpretability. Cell‐type annotation was performed using a two‐step strategy. First, automated annotation was conducted using the SingleR package with Human Primary Cell Atlas (HPCA) data from the celldex package as the reference dataset. Second, canonical cell‐type marker genes were examined using DotPlot and FeaturePlot visualizations to manually review and correct the preliminary annotations; clusters inconsistent with canonical marker‐expression patterns were reassigned accordingly. After cell‐type annotation, tSNE was used for two‐dimensional visualization of the annotated cell populations, and CellChat was applied to infer cell–cell communication networks among the identified cell types. GSE149655 was used solely for exploratory single‐cell characterization and not for validation of patient‐level prognostic performance. For descriptive exploration, the fixed LASSO coefficients derived from the prognostic model were applied to the sample‐level aggregated transcriptomic profile of each sample to calculate a sample‐level score. The four samples (two primary LUAD samples and two normal tissue samples) were divided by the median sample‐level score into higher score and lower score sample groups. Because this dataset contained only four independent samples and the groups could be strongly influenced by tumor‐versus‐normal tissue status, this grouping was used only as an exploratory organizational framework and should not be interpreted as validated prognostic risk stratification. All cells originating from the same sample inherited the corresponding sample‐level score‐group label solely for descriptive visualization and exploratory comparisons of cell–cell communication, cytokine‐related scores, and exhaustion‐related scores in Figure 1D–G.

**Figure 1:**
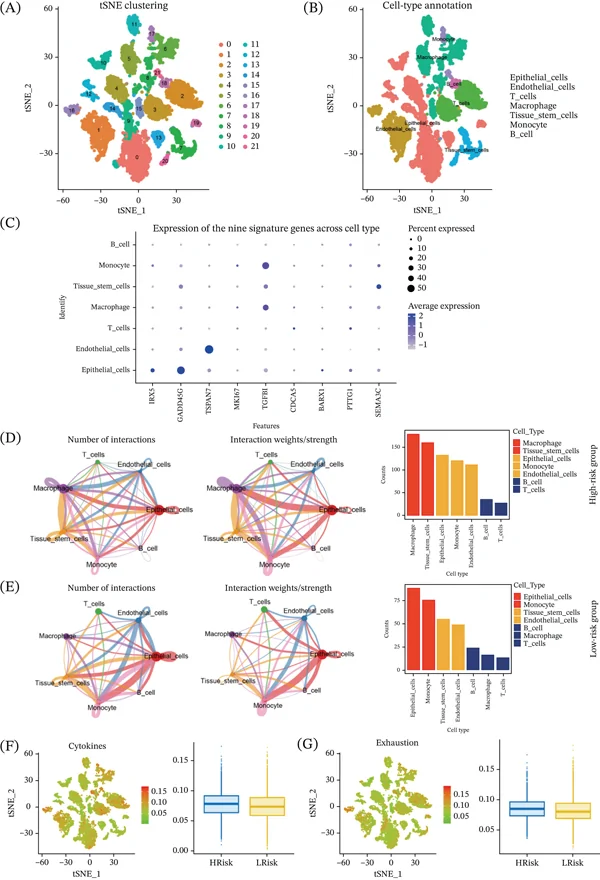
Exploratory single‐cell characterization of the LUAD immune landscape using GSE149655. GSE149655 contains four source samples (two primary LUAD and two normal tissue samples) and was used for exploratory single‐cell characterization rather than patient‐level prognostic validation. Because of the limited number of independent samples, the sample‐level score grouping may be strongly influenced by tumor‐versus‐normal tissue status and should not be interpreted as validated prognostic risk stratification. Single‐cell data were processed with Seurat, cell identities were assigned using SingleR with HPCA data and checked against canonical marker genes, and tSNE was used for visualization. (A) tSNE visualization of 22 cell clusters. (B) Annotation of the clusters into seven major cell types. (C) Dot plot showing the expression of the nine signature genes across the seven annotated cell types. For exploratory score‐group comparisons, one sample‐level nine‐gene score was calculated for each source sample using the fixed model coefficients; the four samples were divided by the median sample‐level score into higher score and lower score sample groups, and all cells from the same sample inherited that sample′s score‐group label. (D,E) CellChat analysis of the number and strength of cell–cell interactions between the higher score and lower score sample groups. (F,G) Descriptive comparison of cytokine‐related and exhaustion‐related scores between the two sample‐level score groups. Panels F and G are presented descriptively; no inferential *p* values are reported because only four independent source samples were available.

### 2.8. Cell Line, Cell Culture, and Knockdown Study

The human LUAD cell line A549 was purchased from the Cell Bank of the Chinese Academy of Sciences. Cells were cultured in high‐glucose DMEM medium supplemented with 10% fetal bovine serum (FBS) and 1% penicillin–streptomycin double antibody, and maintained in a constant temperature and humidity incubator at 37°C with 5%CO_2_. Cells in the logarithmic growth phase were used for subsequent experiments. The knockdown of IRX5 was accomplished with a small interfering RNA (siRNA; HANBIO, China). The transfections were performed with INTERFERin (Polyplus‐transfectionSA) according to the manufacturer′s instructions. The target sequences used for the siRNAs were listed in Table S1.

For the IRX5 knockdown experiments, no formal sample‐size calculation was performed because these experiments were exploratory in vitro functional assays intended to characterize the biological relevance of IRX5 in LUAD cells. Unless otherwise specified, three independent experiments were performed and treated as biological replicates. For the CCK‐8 assay, three replicate wells per group were included within each independent experiment as technical replicates.

### 2.9. Western Blot and Antibodies

Western blot assays were performed by running cell lysates on 10% SDS polyacrylamide gels to separate proteins. The separated proteins were then transferred to a polyvinylidene fluoride membrane via wet transfer at 300 mA for 60 min. After incubating with blocking buffer for 1 h, the indicated antibodies were added to the membrane and incubated at 4°C overnight. The blots were incubated with goat antirabbit IgG H&L (HRP) for 1 h at room temperature. FluorChem HD2 (Protein Sample, United States) was used to detect the proteins via enhanced chemiluminescence. All antibodies used in this study are described in Table S1.

### 2.10. Cell Proliferation, Cell Invasion, and Wound‐Healing Assays

A549 cells were seeded in 96‐well plates at 5000 cells per well, with three technical replicate wells per group in each independent experiment, together with blank zero‐adjustment wells. When cell confluence reached 70%–80%, siRNA transfection was performed as described above. At 0, 24, 48, and 72 h after transfection, 10 *μ*L of CCK‐8 reagent (Beyotime Biotechnology, China) was added to each well. After incubation at 37°C for 1 h, absorbance (OD value) at 490 nm was measured using a microplate reader. Cell proliferation curves were plotted with culture time as the abscissa and OD value as the ordinate to evaluate cell proliferation capacity. All experiments were independently repeated three times; A549 cells were seeded in culture plates, and siRNA transfection was performed at 70%–80% confluence. Cells were used for assays 48 h after transfection. Migration assay: Cells were washed with serum‐free high‐glucose DMEM and digested with trypsin. Cell concentration was adjusted to 1 × 10^5^ cells/mL, and 200 *μ*L of cell suspension was added to the upper chamber of transwell inserts; 600 *μ*L of complete medium with 10% FBS was added to the lower chamber of 24‐well plates, followed by incubation at 37°C with 5% CO_2_ for 24 h. Invasion assay: Matrigel matrix (Corning, United States) was diluted 1:8 with serum‐free medium, and 50 *μ*L was plated into the upper chamber of transwell inserts, then incubated at 37°C for 3 h until solidified. Subsequent procedures were identical to the migration assay. After incubation, transwell inserts were washed twice with PBS. Cells were fixed with 4% paraformaldehyde (Solarbio Science & Technology Co. Ltd., China) at room temperature for 20 min and stained with 0.1% crystal violet (Solarbio Science & Technology Co. Ltd., China) for 15 min. After rising with distilled water, nonmigrated cells on the upper chamber were gently removed with a sterile cotton swab. Five visual fields were randomly selected under an inverted microscope, and the number of migrated cells was counted and averaged to assess migration and invasion capacity. All experiments were independently repeated three times; A549 cells were seeded in sixwell plates at an appropriate density. When cell confluence exceeded 90%, siRNA transfection was performed as described above. Forty‐eight hours after transfection, three parallel scratches of equal width were made vertically with a 200‐*μ*L sterile pipette tip. Cells were washed twice with PBS to remove detached cells, and serum‐free high‐glucose DMEM was added. Scratch morphology was photographed under an inverted microscope at 0, 12, 24, and 36 h after scratching. All experiments were independently repeated three times.

### 2.11. Apoptosis and Cell Cycle Analysis

A549 cells expressing the indicated constructs were treated with the Cell Cycle Staining Kit (Multiscience, China) and an Annexin V, FITC Apoptosis Detection Kit (Dojindo, Japan) according to the manufacturer′s instructions and then analyzed by flow cytometry. The results are presented as the percentage of cells in each phase. The apoptosis and cell‐cycle assays were independently repeated three times.

### 2.12. Statistical Analysis

All statistical analyses were conducted using R Version 4.2. Kaplan–Meier survival curves and log‐rank tests were used for survival analysis. For the IRX5 functional characterization experiments, quantitative data are presented as mean ± standard deviation (SD) from three independent experiments. Comparisons between two groups were performed using two‐tailed unpaired Student′s *t*‐tests. CCK‐8 measurements across multiple time points were analyzed using two‐way analysis of variance (ANOVA) followed by Sidak′s multiple‐comparisons test. A two‐sided *p* value < 0.05 was considered statistically significant. The PHs assumption for the univariate and multivariable Cox regression models was assessed using Schoenfeld residuals; a two‐sided *p* value > 0.05 was considered to indicate no evidence of violation of the PH assumption. For the GSE149655 single‐cell analysis, individual cells were not treated as independent biological replicates. Given the availability of only four independent source samples, the score comparisons shown in Figure 1F,G were presented descriptively without inferential statistical testing or *p* values. Multicollinearity among all covariates included in the multivariable Cox model was assessed using VIFs, with VIF < 10 used as the criterion for absence of severe multicollinearity.

## 3. Results

### 3.1. Construction of Nucleotide Metabolism‐Related Molecular Subtypes and Identification of Subtype‐Associated Differentially Expressed Genes

We extracted gene sets related to nucleotide metabolism with a relevance score > 20 using the GeneCards database (https://www.genecards.org/). Next, we performed consensus clustering of these genes, with the optimal number of clusters determined on the basis of CDF analysis. The CDF delta area curve indicated that the clustering result was more stable when a cluster number of 3 was used (Figure 2A). The clustering heatmap of the three clusters was shown in Figure 2B. Kaplan–Meier survival analysis demonstrated significant differences in OS among the three molecular subtypes, with the C3 subtype showing the poorest prognosis (Figure 2C). Pairwise differential‐expression analyses were then performed for C1 versus C2, C1 versus C3, and C2 versus C3 using the criteria described in the Methods. These comparisons identified 726, 2315, and 726 differentially expressed genes, respectively (Figure S1 and Table S2). The intersection of the three gene sets yielded 152 subtype‐associated differentially expressed genes for subsequent analyses, as shown in the Venn diagram (Figure 2D).

**Figure 2:**
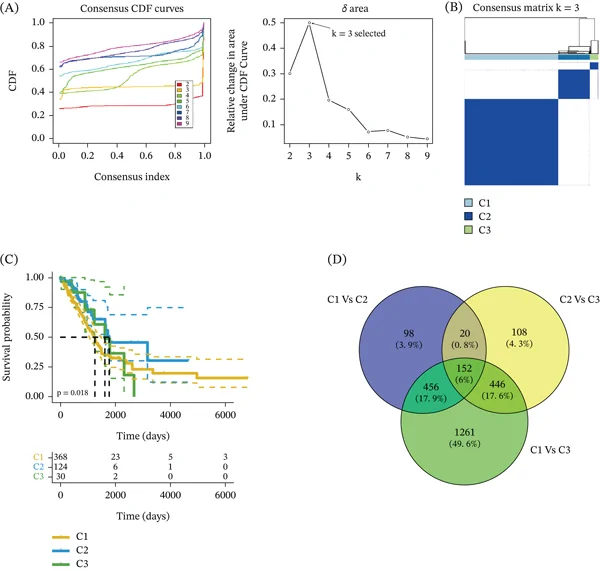
Identification of nucleotide metabolism‐related molecular subtypes in the TCGA‐LUAD cohort. (A) Cumulative distribution function (CDF) curves from consensus clustering of TCGA‐LUAD samples based on nucleotide metabolism‐related genes; the delta area curve supported three stable molecular subtypes (*k* = 3). (B) Consensus clustering heatmap showing the C1, C2, and C3 molecular subtypes. (C) Kaplan–Meier overall survival analysis comparing the three TCGA‐LUAD molecular subtypes. (D) Venn diagram showing the intersection of differentially expressed genes identified from the C1 versus C2, C1 versus C3, and C2 versus C3 comparisons, yielding 152 subtype‐associated differentially expressed genes for subsequent analyses.

### 3.2. Construction of the Subtype‐Derived Prognostic Signature

To investigate the functional characteristics of these genes, we performed enrichment analysis of the 152 subtype‐associated differentially expressed genes using Metascape. Figure 3A presents a bar plot of the significantly enriched terms, whereas Figure 3B shows the network visualization of enriched terms and their relationships. The enrichment results indicated that these genes were predominantly associated with extracellular matrix (ECM), response to nutrients and peptidase regulator activity‐related biological processes. Before prognostic model construction, the TCGA‐LUAD cohort was randomly divided into a training cohort and an internal validation cohort at a ratio of 4:1. All subsequent feature‐selection and model‐fitting procedures were performed exclusively in the training cohort. In the training cohort, univariate Cox regression analysis identified 26 prognostically significant genes with a *p* value < 0.05 (Figure 3C and Table S3). LASSO regression analysis was subsequently performed in the training cohort to further select prognostic genes, resulting in the identification of nine hub genes for construction of the subtype‐derived prognostic signature (Figure 3D–F and Table S4). Based on the coefficients estimated from the training cohort, a subtype‐derived nine‐gene risk score was generated: Risk  score = IRX5 × (−0.128648463) + GADD45G × (−0.077826735) + TSPAN7 × (−0.073307189) + MKI67 × 0.000163894 + TGFBI × 0.02372854 + CDCA5 × 0.035798743 + BARX1 × 0.089405586 + PTTG1 × 0.122970952 + SEMA3C × 0.135467087. The resulting subtype‐derived prognostic signature was subsequently evaluated in the internal validation cohort without additional feature selection or model fitting. The signature significantly stratified patients into low‐ and high‐risk groups, with survival outcomes analyzed using K‐M curves. The OS of the high‐risk group was significantly lower than that of the low‐risk group in both the training and internal validation cohorts (Figure 4A,B). Furthermore, the ROC curves confirmed that the subtype‐derived prognostic signature performed well in predicting the prognosis of LUAD in both the training and internal validation cohorts (Figure 4C,D). Exploratory evaluation was additionally performed in the ICI‐treated GSE135222 and IMvigor210 datasets (Figure S2); notably, IMvigor210 was analyzed only as an exploratory cross‐cancer cohort rather than as direct validation in NSCLC or LUAD. For external evaluation, the fixed nine‐gene model was applied to GSE30219 without additional feature selection or coefficient refitting. For the Kaplan–Meier analysis, patients in GSE30219 were dichotomized using the GSE30219‐specific median risk score, whereas the time‐dependent ROC analysis used the continuous risk score (Figure 4E,F). Accordingly, this analysis evaluates the prognostic performance of the fixed model in an external cohort but does not constitute validation of a prespecified absolute cutoff.

**Figure 3:**
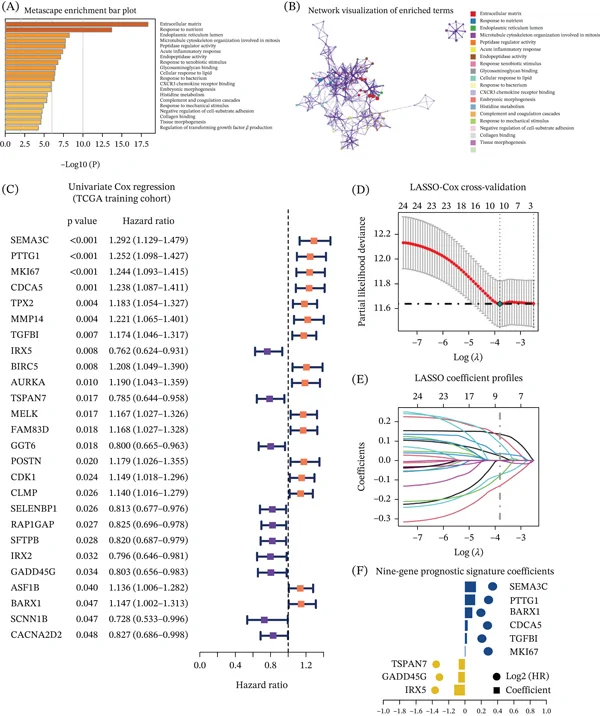
Construction of the nine‐gene subtype‐derived prognostic signature in the TCGA training cohort. (A) Metascape enrichment bar plot of the 152 subtype‐associated differentially expressed genes. (B) Metascape network visualization showing relationships among significantly enriched terms. (C) Univariate Cox proportional hazards regression performed exclusively in the TCGA training cohort; genes with *p* < 0.05 were retained as prognostic candidates. (D,E) LASSO‐Cox regression with an L1 penalty in the training cohort; the optimal penalty parameter was selected by cross‐validation at lambda.min. (F) Coefficient profile of the nine genes retained in the final signature: SEMA3C, PTTG1, BARX1, CDCA5, TGFBI, MKI67, TSPAN7, GADD45G, and IRX5.

**Figure 4:**
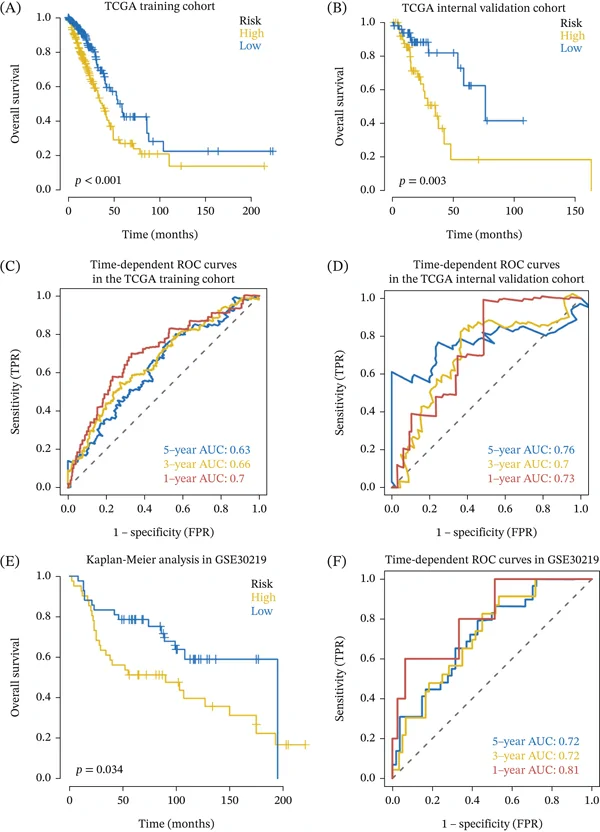
Internal and external validation of the nine‐gene subtype‐derived prognostic signature. (A,B) Kaplan–Meier overall survival curves for the TCGA training cohort and TCGA internal validation cohort after stratification into high‐ and low‐risk groups using the cohort‐specific median risk score within each cohort. (C,D) Time‐dependent ROC curves evaluating the prognostic performance of the fixed nine‐gene signature in the TCGA training and internal validation cohorts. (E,F) Kaplan–Meier survival and time‐dependent ROC analyses in the independent external bulk‐transcriptomic GSE30219 cohort. Feature selection and coefficient estimation were performed only in the TCGA training cohort; the fixed model was applied to the validation cohorts without additional feature selection or model refitting. For the external GSE30219 cohort, the GSE30219‐specific median risk score was likewise used for Kaplan–Meier dichotomization; the training‐cohort median cutoff was not directly applied. Time‐dependent ROC analyses used the continuous risk score and were independent of the dichotomization cutoff. The fixed model coefficients were applied to all validation cohorts without additional feature selection or model refitting.

### 3.3. Independent Prognostic Analysis and Construction of the Nomogram

Univariate and multivariate Cox regression analyses evaluating the nine‐gene risk score together with clinical variables, including age, sex, overall stage, and the individual T, N, and M categories, confirmed that the nine‐gene risk score was an independent prognostic factor (hazard ratio = 4.222, *p* < 0.001) (Figure 5A,B and Table S5). Based on these results, a nomogram integrating the risk score with the same clinical characteristics was constructed for individualized survival prediction (Figure 5C). The calibration curves demonstrated the agreement between the nomogram‐predicted and observed 3‐ and 5‐year OS probabilities (Figure 5D). Because overall stage and the individual T, N, and M categories contain related staging information, we further calculated VIFs for all covariates included in the Cox model. All VIF values were < 10, and no severe multicollinearity was detected. Therefore, overall stage together with the individual T, N, and M categories was retained in the multivariable Cox model and nomogram.

**Figure 5:**
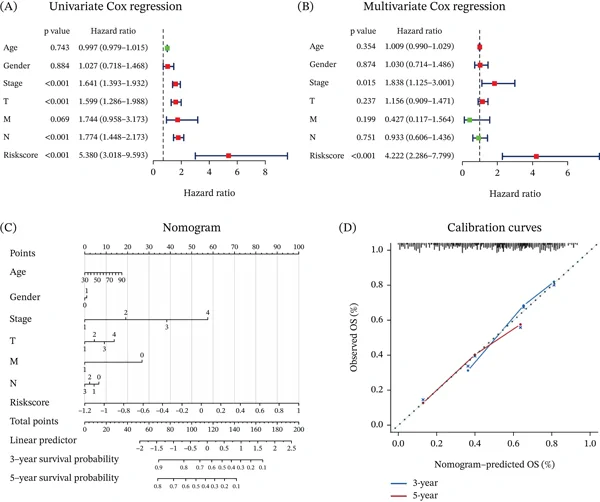
Independent prognostic analysis and construction of the clinical nomogram. (A,B) Univariate and multivariate Cox proportional hazards regression analyses evaluating the nine‐gene risk score together with age, sex, overall stage, and the individual T, N, and M categories. (C) Nomogram integrating the nine‐gene risk score with these clinical variables for individualized survival prediction. (D) Calibration curves comparing nomogram‐predicted and observed 3‐ and 5‐year overall survival probabilities. Potential multicollinearity among all covariates included in the multivariable Cox model was additionally assessed using VIF analysis; all VIF values were < 10.

### 3.4. Estimation of Predicted Drug Response and Molecular Pathways Associated With the Subtype‐Derived Prognostic Signature

To explore potential differences in drug response associated with the subtype‐derived prognostic signature, we used the pRRophetic package to estimate half‐maximal inhibitory concentration (IC50) values in the high‐ and low‐risk groups. Significantly, lower predicted IC50 values for AKT inhibitor VIII, Cisplatin, Dasatinib, Gefitinib and Gemcitabine were observed in the low‐risk group (Figure 6A–E). These IC50 values were computationally estimated rather than experimentally or clinically measured. Subsequently, to investigate the potential molecular mechanisms of this signature, we performed GSEA using the KEGG LEGACY gene set collection from the C2:CP collection of MSigDB Human v2022.1.Hs. The enrichment analysis revealed that alpha linolenic acid metabolism, arachidonic acid metabolism, fatty acid metabolism, and linoleic acid metabolism signaling pathways were significantly enriched in the high‐risk group, whereas cell cycle and ubiquitin‐mediated proteolysis pathways were enriched in the low‐risk group (Figure 6F).

**Figure 6:**
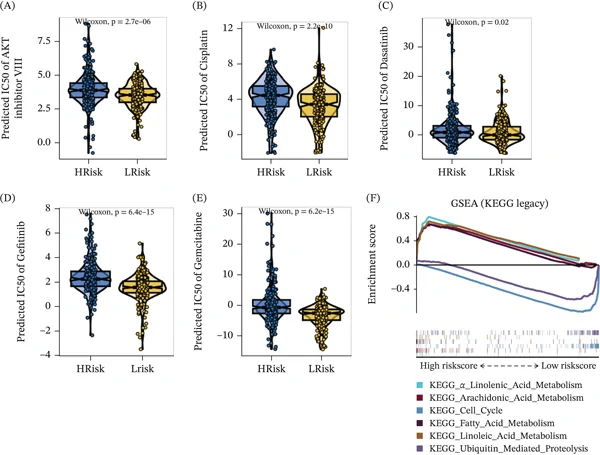
Computationally predicted drug‐response patterns and pathway differences associated with the nine‐gene risk groups. (A–E) pRRophetic‐based computational estimates of half‐maximal inhibitory concentration (IC50) for AKT inhibitor VIII, cisplatin, dasatinib, gefitinib, and gemcitabine in the TCGA high‐ and low‐risk groups; these values represent bioinformatic predictions rather than clinically measured drug responses. (F) Gene set enrichment analysis performed with GSEA software Version 4.3.2 using the KEGG LEGACY gene‐set collection from C2:CP, MSigDB Human v2022.1.Hs, showing pathway‐level differences between the high‐ and low‐risk groups.

### 3.5. The Mutational Landscape and Immune Features Associated With the Subtype‐Derived Prognostic Signature

The mutational landscape for patients within high‐ and low‐risk groups was further explored. A heatmap was generated to visualize the main mutation sites and ratios between different groups. It indicated that patients in the high‐risk group exhibited a significantly higher mutation ratio for genes such as TP53 compared with the low‐risk group (Figure 7A). Meanwhile, in consideration of subgroups with specific clinical significance, such as EGFR or ALK‐positive patients, smokers and patients in different age groups, we found that patients with ALK‐positive status tend to exhibit relatively higher risk scores compared with ALK‐negative patients (Figure S3). On the other hand, the TME plays an important role in various stages of tumor generation, metastasis, and evasion of immune monitoring and treatment. The TME primarily comprises cancer‐associated fibroblasts (CAFs), ECM, tumor blood vessels, and nontumor cells [23]. We further calculated the proportions of different immune cells among the LUAD patients in both groups, the results of which are shown in Figure 7B. We then investigated the relationship between immune infiltration and the subtype‐derived prognostic signature in the TME. As a result, CD4 memory T cells, M0 macrophages, and M1 macrophages were significantly increased in the high‐risk group. Conversely, naïve B cells, plasma cells, and activated NK cells were significantly decreased in the high‐risk group (Figure 7C).

**Figure 7:**
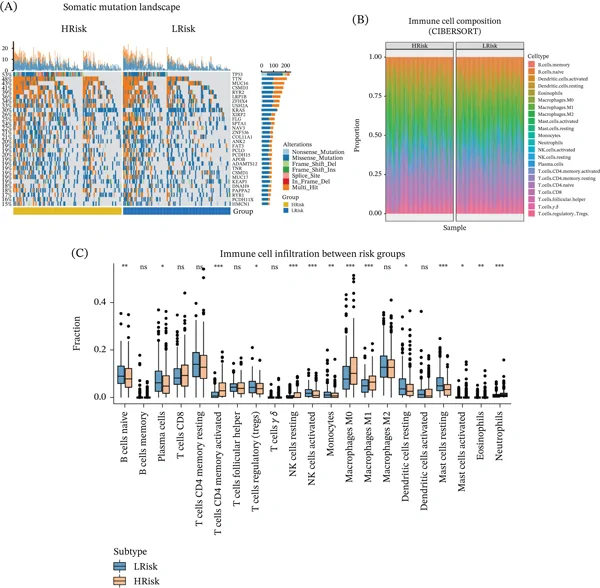
Somatic mutation and immune‐infiltration features associated with the nine‐gene risk groups in TCGA‐LUAD. (A) Comparison of the somatic mutation landscape and mutation frequencies between TCGA‐LUAD patients in the high‐ and low‐risk groups. (B) Relative immune‐cell composition estimated from bulk transcriptomic data using CIBERSORT. (C) Comparison of CIBERSORT‐estimated immune‐cell fractions between the two risk groups.  ^∗^ *p* < 0.05;  ^∗∗^ *p* < 0.01; and  ^∗∗∗^ *p* < 0.001.

### 3.6. Exploratory Single‐Cell Characterization of the Immune Landscape in LUAD

The GSE149655 dataset was analyzed as an exploratory single‐cell dataset to characterize the cell‐type distribution and expression patterns of signature genes, rather than to validate patient‐level prognosis. Because the dataset included only four independent source samples (two primary LUAD and two normal tissue samples), the sample‐level score grouping was considered exploratory and may reflect tumor‐versus‐normal tissue differences rather than meaningful prognostic risk stratification. Using the Seurat clustering workflow, 22 cell clusters were identified and subsequently visualized by tSNE (Figure 1A). Cell identities were initially assigned using SingleR with the HPCA reference and were further reviewed against canonical cell‐type marker genes, resulting in seven major cell categories: epithelial cells, endothelial cells, T cells, macrophages, tissue stem cells, monocytes, and B cells (Figure 1B). The expression profiles of selected key molecules across these cell types are present in Figures 1C and S4. To explore the underlying intercellular communication context, we applied the CellChat algorithm. In the higher score sample group, the inferred communication network appeared more extensive, with macrophages showing prominent potential interactions with other cell types; in the lower score sample group, epithelial cells appeared to show more prominent interactions. SEMA3C also showed more extensive inferred interactions with multiple receptors in the higher score sample group (Figures 1D,E, S5, and S6). Cytokine‐ and exhaustion‐related scores were examined descriptively and appeared higher in the higher score sample group (Figure 1F,G); no inferential *p* values were reported for this comparison. Meanwhile, the associations of signature hub molecules and the scores were analyzed and depicted in Figure S7.

### 3.7. Gene Expression Assessment and Functional Characterization of IRX5

To elucidate the mRNA expression pattern of the nine genes included in the subtype‐derived prognostic signature, LUAD, CDCA5, PTTG1, MKI67, TGFBI, BARX1, GADD45G, and IRX5 exhibited a highly cancer‐specific expression pattern, whereas TSPAN7 and SEMA3C showed the opposite trend according to an analysis of the Gene Expression Profiling Interactive Analysis database (http://gepia.cancer-pku.cn/) (Figure S8). Then, IRX5 was knocked down in A549 cells, as shown in Figure 8A. CCK‐8 assays showed that the knockdown of IRX5 significantly inhibited the proliferation of A549 cells (Figure 8B). Transwell and wound‐healing assays uncovered that the knockdown of IRX5 suppressed the invasiveness and migration capability of A549 cells (Figure 8C,D). Furthermore, flow cytometry analysis demonstrated a significantly increased proportion of apoptotic cells and decreased the proportion of cells in S and G2‐M phase following the knockdown of IRX5 (Figure 8E,F).

**Figure 8:**
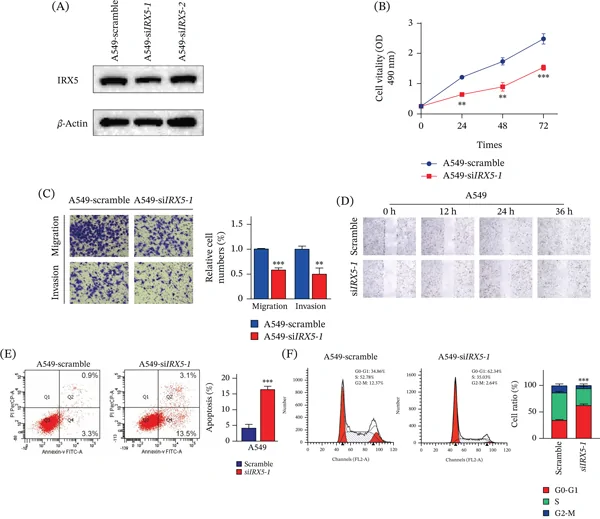
In vitro functional characterization of IRX5 in A549 LUAD cells. IRX5 was silenced by siRNA, and the control and IRX5‐knockdown groups were compared in three independent experiments. Quantitative data are presented as mean ± SD. Two‐group comparisons were analyzed using two‐tailed unpaired Student′s *t*‐tests; CCK‐8 measurements across multiple time points were analyzed using two‐way ANOVA followed by Sidak′s multiple‐comparisons test.  ^∗^ *p* < 0.05;  ^∗∗^ *p* < 0.01;  ^∗∗∗^ *p* < 0.001; and  ^∗∗∗∗^ *p* < 0.0001. (A) Western blot verification of IRX5 knockdown efficiency. (B) CCK‐8 assay evaluating cell proliferation. (C) Transwell assays evaluating migration and invasion. (D) Wound‐healing assay evaluating cell migration. (E) Flow‐cytometric analysis of apoptosis. (F) Flow‐cytometric analysis of cell‐cycle distribution.

## 4. Discussion

Lung cancer is extremely aggressive and can be divided into small cell lung cancer and NSCLC; LUAD represents the most common histological subtype of NSCLC; therefore, exploring effective prognostic indicators for this disease is clinically important [24, 25]. With the advancements in sequencing technology, the traditional prognostic assessment system has failed to facilitate precision medicine [26, 27]. There is an urgent need to identify sensitive and specific biomarkers to help with clinical diagnosis [28, 29]. Recent research has demonstrated that metabolic aberration is an important feature of tumors and that aberrant nucleotide metabolism can suppress the TME immune response to promote the progression of tumors [30–32]. In addition, the altered metabolic environment can also affect the traditional therapeutic and immune responses of tumors and may cause immune escape [33–35]. Therefore, an in‐depth study of the potential value of metabolism‐related genes in cancer is of clinical significance.

In our study, we analyzed the transcriptome sequencing data of LUAD patients via a bioinformatics approach. Potential prognostic genes were mined, and a novel subtype‐derived prognostic signature was constructed. The subtype‐derived prognostic signature consisted of nine hub molecules, including SEMA3C, PTTG1, BARX1, CDCA5, TGFBI, MKI67, TSPAN7, GADD45G, and IRX5. Importantly, this signature was derived from differentially expressed genes among molecular subtypes defined using nucleotide metabolism‐related genes; therefore, it should be interpreted as a subtype‐derived prognostic signature based on nucleotide metabolism‐related molecular classification, rather than as evidence that all nine genes directly participate in nucleotide metabolism pathways. Several hub molecules have been proven to be associated with the development and progression of LUAD. For example, PTTG1 was one type of DNA repair‐related gene and might promote progression of LUAD via the P53 signaling pathway [36]. BARX1 could repress FOXF1 expression and activated Wnt/*β*‐catenin signaling pathway to drive LUAD [37]. CDCA5 regulated the cell cycle of NSCLC cells by mediating the P53‐P21 signaling pathway, participating in the development and progression of NSCLC patients [38]. These studies highlight the significance of these molecules in LUAD and their potential as prognostic factors. Furthermore, the subtype‐derived prognostic signature has been shown to exhibit good prediction efficiency in both TCGA and GEO cohorts. We found that integrating these nine molecules into one parameter could improve the accuracy of prognosis prediction. The risk score calculated based on this signature shows potential in distinguishing the prognosis of LUAD patients. Meanwhile, independent prospective or retrospective clinical cohorts were needed to avoid selection bias and inconsistent data quality.

Although overall stage and the individual T, N, and M categories are clinically related, they were retained in the Cox model and nomogram because they provide complementary staging information at different levels. T, N, and M describe local tumor extent, regional lymph‐node involvement, and distant metastatic status, whereas overall stage integrates these components into a comprehensive clinical classification. We further assessed potential multicollinearity by calculating VIFs for all covariates included in the Cox model; all VIF values were < 10, indicating that no severe multicollinearity was detected. Retaining both the integrated overall stage and the individual TNM components preserves both summary and component‐level staging information and may improve the practical interpretability of the prognostic nomogram in clinical settings with different levels of staging information available.

Chemotherapy remains the primary and effective treatment for LUAD patients [39, 40]. However, the efficacy of chemotherapy is variable. To explore the potential association between the subtype‐derived prognostic signature and drug response, we used pRRophetic to estimate IC50 values for selected anticancer agents. The low‐risk group showed lower predicted IC50 values for AKT inhibitor VIII, Cisplatin, Dasatinib, Gefitinib, and Gemcitabine. However, these results represent computational predictions and should not be interpreted as direct evidence of clinical drug sensitivity or treatment benefit. Further validation in cellular and animal models, as well as clinical studies, is required. In order to further investigate the potential molecular mechanisms, we employed GSEA analysis. The pathways of alpha linolenic acid metabolism, arachidonic acid metabolism, fatty acid metabolism, and linoleic acid metabolism were notably enriched in the high‐risk group, as cell cycle and ubiquitin‐mediated proteolysis in the low‐risk group. The current studies revealed that these pathways play crucial roles in the development and metastasis of LUAD [41–45]. In addition, the TME plays a crucial role in the antitumor response and can significantly affect patient prognosis [46–48]. We investigated the relationship between the subtype‐derived prognostic signature and the TME. We observed a significant increase in the infiltration of CD4 memory T cells, M0 macrophages, and M1 macrophages in the high‐risk group, as well as a decrease in B cells naïve, plasma cells, and NK cells. This pattern suggests an overall trend toward immune suppression. In the exploratory single‐cell dataset, the higher score sample group appeared to show stronger macrophage‐related communication and higher cytokine‐ and exhaustion‐related scores. These immune‐related features have been associated with tumor progression, abnormal activation of the immune system, and inflammatory responses [49]. However, because GSE149655 included only two LUAD and two normal tissue samples, these observations may be strongly influenced by tumor‐versus‐normal tissue differences and should not be interpreted as evidence of prognostic risk stratification.

Notably, IRX5 had a negative coefficient in the multigene risk model. This coefficient represents its adjusted prognostic contribution within the entire signature and should not be simply interpreted as the isolated biological function of IRX5. The negative coefficient may also be influenced by the multigene model structure, correlations among gene‐expression predictors, tumor heterogeneity, cell‐type composition, and the clinical context of the analyzed cohort; therefore, the coefficient direction should not be interpreted as a direct measure of the isolated biological effect of IRX5. Given this complexity, we selected IRX5 for functional characterization to explore its biological relevance in LUAD cells. We designed two specific siRNAs targeting IRX5 and successfully knocked down its expression in A549 LUAD cells, which was verified by western blotting. A series of in vitro experiments showed that IRX5 knockdown inhibited the proliferation, migration and invasion of A549 LUAD cells, promoted apoptosis, and induced cell‐cycle arrest in the G0/G1 phase. These results suggest that IRX5 may contribute to malignant phenotypes of LUAD cells by affecting cell‐cycle progression and apoptosis. As a member of the subtype‐derived prognostic signature, the IRX5 knockdown experiments support the functional relevance of IRX5 in LUAD cells. However, these functional assays should be interpreted as evidence supporting the biological relevance of IRX5, rather than as direct validation of the negative coefficient of IRX5 in the multigene prognostic model. Moreover, IRX5 may represent a potential molecule worthy of further mechanistic investigation. In addition, the altered expression pattern of IRX5 in LUAD tissues based on TCGA data further suggests its potential involvement in LUAD biology.

However, there are some limitations to our study: All the RNA sequencing data and clinical information were obtained from public databases, such as the TCGA and GEO databases; the in vitro functional experiments of IRX5 were only conducted in cell lines, and the in vivo experimental verification using animal models has not been completed. Furthermore, the pRRophetic‐derived IC50 values represent computational estimates rather than clinically measured drug responses and require further experimental and clinical validation. The single‐cell analysis was additionally limited by the very small number of independent source samples (two LUAD and two normal tissues); therefore, the sample‐level score grouping should be regarded as descriptive and exploratory, and larger single‐cell cohorts with adequate numbers of independent tumor samples and clinical outcome data are required to evaluate prognostic relevance. We plan to perform prospective clinical research, in vivo animal experiments and further genetic functional research on other hub genes in the signature in the future to further verify the predictive significance of this subtype‐derived prognostic signature and explore the molecular mechanisms of each hub gene in LUAD.

## 5. Conclusions

In this study, the nine‐gene subtype‐derived prognostic signature based on nucleotide metabolism‐related molecular classification constructed from bulk transcriptomic data and further characterized using single‐cell transcriptomic data showed potential for stratifying survival outcomes in LUAD and was associated with computationally predicted drug‐response patterns. This signature may serve as a candidate prognostic biomarker, but further prospective validation is required to assess its utility in clinical decision‐making.

**Nomenclature**
AUC: area under the curve
CAFs: cancer‐associated fibroblasts
CDF: cumulative distribution function
ECM: extracellular matrix
GO: gene ontology
GSEA: gene set enrichment analysis
KEGG: Kyoto Encyclopedia of Genes and Genomes
LASSO: least absolute shrinkage and selection operator
LUAD: lung adenocarcinoma
LUSC: lung squamous cell carcinoma
OS: overall survival
ROC: receiver operating characteristic
TME: tumor microenvironment
tSNE: t‐distributed stochastic neighbor embedding
VIF: variance inflation factor

## Author Contributions

Conception and design: Jian Zhang, Xue Bai, Changguo Shi, and Yue Cui. Data acquisition, assembly, and experiments: Yue Shi, Lin Zhao, and Yali Li. Data analysis and interpretation: Fangyu Cai, Kun Wang, Benkun Liu, and Fucheng Zhou. Writing—original draft preparation and revision: Yue Shi and Shanshan Li. Yue Shi, Shanshan Li, Lin Zhao, and Yali Li contributed equally to this work.

## Funding

This study was supported by National Natural Science Foundation of China (82303616); Joint Fund Cultivation Project of Heilongjiang Provincial Natural Science Foundation (PL2024H171); Haiyan Science Foundation of Harbin Medical University Cancer Hospital (JJYQ2024‐07); Clinical Application Research of Single‐port Thoracoscopic Surgery (W2017ZWS02); Climbing Fund of Harbin Medical University Cancer Hospital (PDTS2024A‐05); and Individualized and precise treatment of lung cancer (Nn10py2017‐04).

## Disclosure

An earlier version of this manuscript was posted as a preprint on Research Square [50]. All authors have reviewed and approved the final version of the manuscript and consent to its publication.

## Ethics Statement

This study did not involve newly recruited human participants or animal experiments. The transcriptomic, clinical, and single‐cell sequencing data used in this study were obtained from public databases and were deidentified. The in vitro experiments were performed using commercially available LUAD cell lines and did not require ethical approval for human participants.

## Consent

The study did not involve human participants, and all data used are anonymized and publicly accessible.

## Conflicts of Interest

The authors declare no conflicts of interest.

## Supporting Information

Additional supporting information can be found online in the Supporting Information section.

## Supporting information


**Supporting Information 1** Figure S1: Pairwise differential‐expression patterns among the TCGA‐LUAD molecular subtypes. (A) Heatmap of 726 genes differentially expressed between C1 and C2. (B) Heatmap of 726 genes differentially expressed between C2 and C3. (C) Heatmap of 2315 genes differentially expressed between C1 and C3. These subtype‐associated gene sets were used to derive the 152 subtype‐associated differentially expressed genes shown in Figure 2D.

**Supporting Information 2** Figure S2: Exploratory evaluation of the nine‐gene subtype‐derived prognostic signature in immune‐checkpoint‐inhibitor–treated cohorts. (A,B) Time‐dependent ROC analyses in GSE135222, an NSCLC immunotherapy cohort, and IMvigor210, a metastatic urothelial carcinoma cohort. The fixed nine‐gene model was applied without model refitting. IMvigor210 was included only as an exploratory cross‐cancer immunotherapy dataset and was not considered direct validation in NSCLC or LUAD.

**Supporting Information 3** Figure S3: Clinical subgroup comparisons of the nine‐gene risk score in TCGA‐LUAD. Boxplots show risk‐score distributions according to (A) ALK status, (B) EGFR status, (C) smoking history, and (D) age group.

**Supporting Information 4** Figure S4: Single‐cell expression of the nine signature genes across the seven annotated cell types in GSE149655. Cell populations were identified using the Seurat/SingleR workflow described in the methods; this analysis was exploratory and was not used for patient‐level prognostic validation.

**Supporting Information 5** Figure S5: CellChat‐based comparison of intercellular communication between the sample‐level higher score and lower score groups in GSE149655. (A) Overall number and strength of inferred cell–cell interactions. (B) Differential interaction networks. (C) Sender–receiver heatmaps summarizing differential communication patterns. (D) Differential analysis of major signaling pathways. Score‐group labels were assigned at the source‐sample level and inherited by all cells from the same sample for exploratory descriptive comparisons.

**Supporting Information 6** Figure S6: CellChat ligand‐receptor analysis in GSE149655. (A,B) Representative inferred ligand–receptor interactions sample‐level higher score and lower score groups; solid lines indicate inferred ligand–receptor connections. The comparison used the same exploratory sample‐level score‐group labels described for Figure 1.

**Supporting Information 7** Figure S7: Associations between signature‐gene expression and immune‐related scores in the GSE149655 single‐cell dataset. (A) Associations of PTTG1, TGFBI, MKI67, TSPAN7, and IRX5 with cytokine‐related scores. (B) Associations of SEMA3C, PTTG1, TGFBI, and TSPAN7 with exhaustion‐related scores. These analyses used the exploratory single‐cell framework described in Section 2.7.

**Supporting Information 8** Figure S8: Expression patterns of the nine signature genes in LUAD and normal tissues obtained from the Gene Expression Profiling Interactive Analysis (GEPIA) database, used as an additional descriptive assessment of signature‐gene expression.

**Supporting Information 9** Table S1: IRX5 was inhibited using siRNA targeting the human IRX5 gene.

**Supporting Information 10** Table S2: Detailed subtype‐associated differentially expressed genes identified from pairwise comparisons among the C1, C2 and C3 molecular subtypes.

**Supporting Information 11** Table S3: Detailed information on the 26 prognostically significant genes identified in the TCGA training cohort.

**Supporting Information 12** Table S4: Detailed information on the nine hub genes and corresponding coefficients derived from the TCGA training cohort.

**Supporting Information 13** Table S5: The table of univariate and multivariate Cox regression analyses.

**Supporting Information 14** File S1: STROBE checklist.

## Data Availability

The data that support the findings of this study are available from the corresponding authors upon reasonable request.
