# Supplementary material for: Identification of a Novel Subtype‐Derived Prognostic Signature Based on Nucleotide Metabolism‐Related Molecular Classification for Predicting Lung Adenocarcinoma Prognosis
Source: Int J Genomics. 2026 Oct 2;2026:4944325. doi: 10.1155/ijog/4944325 (PMC13633477; doi:10.1155/ijog/4944325)
Supplement: Supplementary file 1 [file IJOG-2026-4944325-s007.docx]

STROBE Statement—checklist of items that should be included in reports of observational studies

|  | Item No | Recommendation |
| --- | --- | --- |
| **Title and abstract** | 1 | (a) The study is a retrospective cross-sectional observational study based on public multi-omics databases combined with in vitro cell functional validation. |
|  |  | (b) We mined nucleotide metabolism-related prognostic genes from TCGA and GEO LUAD transcriptomic datasets, constructed a 9-gene risk signature, and analyzed its correlation with tumor mutation landscape, immune infiltration, intercellular communication and drug response. Single-cell transcriptome data was used to dissect tumor microenvironment features. Knockdown of IRX5 in A549 cells was performed to verify its oncogenic phenotypes in vitro. |
| Introduction | | |
| Background/rationale | 2 | Lung adenocarcinoma (LUAD) exhibits prominent clinical heterogeneity. Nucleotide metabolic reprogramming drives tumor progression and reshapes the tumor immune microenvironment, yet its prognostic value for LUAD remains poorly characterized. Public TCGA/GEO multi-omics cohorts provide large-scale de-identified clinical transcriptomic data, which enables systematic screening of metabolism-associated prognostic biomarkers for LUAD. |
| Objectives | 3 | Primary objectives: 1) Identify nucleotide metabolism-related genes and establish a robust nine-gene prognostic risk signature for LUAD; 2) Explore the association between risk stratification, genomic mutation, immune landscape, cell-cell communication and chemotherapeutic/immunotherapy sensitivity; 3) Validate the biological function of hub gene IRX5 in LUAD cell line A549.Prespecified hypothesis: LUAD patients with high nucleotide metabolism risk scores harbor immunosuppressive microenvironments, inferior overall survival and weaker response to standard anti-tumor agents. |
| Methods | | |
| Study design | 4 | The clinical cohort part adopts a retrospective cross-sectional design using de-identified public transcriptomic, single-cell and clinical data from TCGA and GEO databases. Supplementary in vitro functional experiments were conducted on human LUAD A549 cell line. |
| Setting | 5 | All clinical data were downloaded from open-access public databases (TCGA-LUAD, GSE30219, GSE149655, GSE135222, IMvigor210). No prospective patient recruitment was performed in our institution. Original sample collection time points were archived records of previous multi-center clinical trials; data sorting and bioinformatic analyses were completed during 2024–2025. No prospective follow-up was carried out by our team; survival endpoints were extracted from pre-stored database clinical annotations. |
| Participants | 6 | Cross-sectional study eligibility criteria: patients pathologically diagnosed with lung adenocarcinoma, with complete transcriptomic profiles and overall survival (OS) records. Exclusion criteria: cases missing survival data or matched gene expression matrix. Data sources: TCGA-LUAD cohort (n=521), external validation cohort GSE30219, single-cell cohort GSE149655 (4 samples), immunotherapy cohorts GSE135222 & IMvigor210. No case-control or matched cohort design was adopted. |
| Variables | 7 | Outcome variable: overall survival (OS) defined by survival/death status recorded in database files.Predictors: expression levels of 152 nucleotide metabolism-related genes, nine-gene risk score.Potential confounders: age, gender, TNM stage, smoking history, EGFR/ALK/TP53 mutation status.Effect modifiers: immune cell infiltration proportions, tumor metabolic pathway activity.Diagnostic criteria for LUAD were sourced from original pathology reports uploaded to public databases. |
| Data sources/ measurement | 8* | Bulk RNA-seq data: normalized FPKM matrix retrieved from TCGA GDC and GEO repositories.Single-cell RNA-seq: raw data from GSE149655, processed and quality-controlled via Seurat package.Clinical, mutational and drug response information: matched supplementary clinical metadata attached to each dataset.Nucleotide metabolism gene set was obtained from GeneCards database.Uniform normalization and quality control pipelines were applied across all datasets to guarantee method comparability. |
| Bias | 9 | Selection bias: strict inclusion/exclusion thresholds were applied to filter incomplete samples; independent training and external validation cohorts were used to reduce single-dataset bias.Measurement bias: standardized bioinformatic pipelines were unified for all transcriptome and single-cell data processing.Confounding bias: univariate and multivariate Cox regression models were adjusted for age and tumor stage; stratified subgroup analyses were performed to eliminate confounding interference. |
| Study size | 10 | Sample size was determined by the number of qualified de-identified cases available in public databases. The TCGA LUAD cohort was randomly split into training (4/5) and internal validation (1/5) sets; GSE30219 served as independent external validation. No pre-study sample size calculation was conducted for this retrospective database analysis. |
| Quantitative variables | 11 | Gene expression values, risk scores, immune cell proportions and predicted IC50 values were continuous variables. The median risk score of the TCGA training cohort was set as the cut-off value to dichotomize patients into high-risk and low-risk groups, consistent with standard stratification strategies for tumor prognostic signatures. |
| Statistical methods | 12 | 12(a) All statistical analyses were performed with R v4.2. Cox proportional hazard models (univariate & multivariate) adjusted for age and TNM stage were used to identify independent prognostic factors. Kaplan-Meier log-rank test was used for survival comparison. |
|  |  | (b) Subgroup analyses stratified by mutation status, smoking history and immunotherapy cohorts; GSEA was applied to detect pathway interactions between risk subgroups. |
|  |  | (c) Cases with missing clinical or expression data were excluded, complete-case analysis was adopted. |
|  |  | (d) Not applicable; no matched design or active prospective follow-up loss occurred in this cross-sectional database study. |
|  |  | (e) No sensitivity analyses were performed in this research. |

Continued on next page

| Results | | |
| --- | --- | --- |
| Participants | 13* | (a) Initial TCGA-LUAD dataset contained 594 patients; 73 cases with incomplete survival or expression data were excluded, leaving 521 eligible samples. The 521 patients were randomly allocated to training set (n=417) and internal validation set (n=104). All qualified samples from GSE30219, GSE149655, GSE135222 and IMvigor210 were included in subsequent analyses. |
|  |  | (b) Exclusion reason: lack of complete overall survival follow-up data or matched transcriptomic profiles. |
|  |  | (c) A participant screening flowchart can be generated for visualization. |
| Descriptive data | 14* | (a) Baseline characteristics including age, gender, TNM stage, smoking history, TP53/EGFR/ALK mutation distribution of TCGA cohort were summarized. Baseline gene expression, risk scores and immune infiltration levels of high/low-risk subgroups were reported. |
|  |  | (b) Only a tiny fraction of samples contained partial missing clinical records and were directly excluded from analysis. |
|  |  | (c) Median overall survival time of each subgroup was summarized based on database archived follow-up records. |
| Outcome data | 15* | (a) Both unadjusted and confounder-adjusted hazard ratios (HR=4.222, P<0.001) with 95% CIs were presented; adjusted confounders included patient age and TNM stage. |
|  |  | (b) Continuous risk score was dichotomized at the median value of TCGA training cohort. |
|  |  | (c) Absolute risk transformation from relative risk estimates was not performed. |
| Main results | 16 | (*a*) Give unadjusted estimates and, if applicable, confounder-adjusted estimates and their precision (eg, 95% confidence interval). Make clear which confounders were adjusted for and why they were included |
|  |  | (*b*) Report category boundaries when continuous variables were categorized |
|  |  | (*c*) If relevant, consider translating estimates of relative risk into absolute risk for a meaningful time period |
| Other analyses | 17 | Supplementary analyses included consensus molecular subtype clustering, CIBERSORT immune infiltration estimation, single-cell cell-cell communication analysis, GSEA pathway enrichment, drug sensitivity prediction, and in vitro A549 cell functional experiments. No sensitivity analyses were conducted. |
| Discussion | | |
| Key results | 18 | In line with predefined research objectives, our nine-gene nucleotide metabolism signature reliably predicts LUAD overall survival across multiple independent cohorts. High-risk patients displayed elevated TP53 mutation frequency, immunosuppressive tumor microenvironment and poorer response to chemotherapeutic agents. Single-cell data revealed enhanced macrophage-mediated intercellular crosstalk in high-risk populations. In vitro IRX5 knockdown suppressed proliferation, migration and invasion, induced G0/G1 arrest and apoptosis in A549 cells. |
| Limitations | 19 | Limitation 1: All clinical data were retrospective de-identified records from public databases, lacking prospective independent clinical cohort validation, which may introduce inherent selection bias.Limitation 2: Functional validation was only conducted in single A549 cell line; in vivo animal model verification was absent.Limitation 3: Drug sensitivity results were purely computational predictions via pRRophetic algorithm without cellular drug response validation.Potential bias direction: Heterogeneous multi-center source of database samples may slightly attenuate the predictive performance of the risk signature. |
| Interpretation | 20 | Comprehensive interpretation integrating multi-omics data and in vitro cell results: Nucleotide metabolic reprogramming promotes malignant progression and immune escape in LUAD; the established risk signature serves as a candidate biomarker for prognosis stratification and individualized therapeutic guidance. Conclusions are preliminary and require prospective clinical cohorts and in vivo animal experiments for further confirmation. |
| Generalisability | 21 | All clinical samples were sourced from Western multi-center public datasets. The predictive value of this signature may not be fully generalizable to Asian LUAD populations. The model is only applicable to pathologically confirmed primary lung adenocarcinoma, not lung squamous cell carcinoma or small cell lung cancer. |
| Other information | | |
| Funding | 22 | This work was supported by the National Natural Science Foundation of China (Grant No. 82303616), Joint Cultivation Project of Heilongjiang Provincial Natural Science Foundation (PL2024H171), Haiyan Research Fund of Harbin Medical University Cancer Hospital (JJYQ2024-07), Climbing Fund of Harbin Medical University Cancer Hospital (PDTS2024A-05), and Lung Cancer Individualized Precision Treatment Program (Nn10py2017-04). All funding bodies had no role in study design, data collection, analysis, manuscript writing or submission. |

*Give information separately for cases and controls in case-control studies and, if applicable, for exposed and unexposed groups in cohort and cross-sectional studies.

**Note:** An Explanation and Elaboration article discusses each checklist item and gives methodological background and published examples of transparent reporting. The STROBE checklist is best used in conjunction with this article (freely available on the Web sites of PLoS Medicine at http://www.plosmedicine.org/, Annals of Internal Medicine at http://www.annals.org/, and Epidemiology at http://www.epidem.com/). Information on the STROBE Initiative is available at www.strobe-statement.org.
